# (*E*)-4-Amino-*N*′-(5-chloro-2-hy­droxy­benzyl­idene)benzohydrazide

**DOI:** 10.1107/S160053681201803X

**Published:** 2012-04-28

**Authors:** Zhong-Feng Shi, Jia-Ming Li

**Affiliations:** a College of Chemistry and Chemical Engineering, Qinzhou University, Qinzhou, Guangxi 535000, People’s Republic of China; b Guangxi Key Laboratory of Petrochemical Resource Processing and Process Intensification Technology, Guangxi University, Nanning, Guangxi 530004, People’s Republic of China

## Abstract

The title compound, C_14_H_12_ClN_3_O_2_, displays an *E* conformation with respect to the C=N double bond. The dihedral angle between the benzene rings is 41.3 (5)°. The mol­ecular structure is stabilized by an intra­molecular O—H⋯N hydrogen bond. In the crystal, N—H⋯O and weak N—H⋯Cl hydrogen bonds link the mol­ecules into a three-dimensional architecture. In addition, there are weak C—H⋯π stacking inter­actions.

## Related literature

For the biological properties of Schiff base and hydrazone compounds, see: Kucukguzel *et al.* (2006 ▶); Khattab (2005 ▶); Karthikeyan *et al.* (2006 ▶). For closely related structures and background references, see: Bernhardt *et al.* (2003 ▶, 2005 ▶); Armstrong *et al.* (2003 ▶); Cao (2009 ▶); Yang (2009 ▶); Zhou & Yang (2010 ▶); Zhang *et al.*, (2009 ▶).
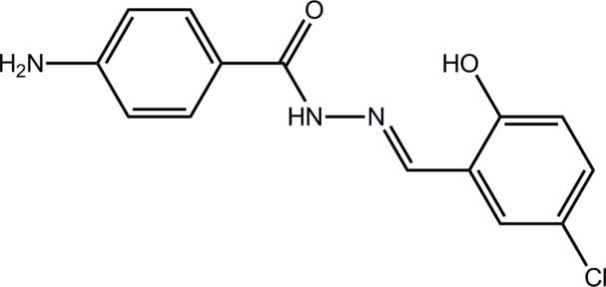


## Experimental

### 

#### Crystal data

C_14_H_12_ClN_3_O_2_
*M* *_r_* = 289.72
Orthorhombic, 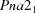

*a* = 9.3375 (16) Å
*b* = 9.7194 (16) Å
*c* = 14.214 (3) Å
*V* = 1290.0 (4) Å^3^
*Z* = 4
Mo *K*α radiation
μ = 0.30 mm^−1^
*T* = 296 K
0.20 × 0.15 × 0.10 mm

#### Data collection

Bruker SMART CCD area-detector diffractometer
Absorption correction: multi-scan (*SADABS*; Sheldrick, 1996 ▶) *T*_min_ = 0.947, *T*_max_ = 0.970
8516 measured reflections
2944 independent reflections
2866 reflections with *I* > 2σ(*I*)
*R*_int_ = 0.038

#### Refinement

*R*[*F* ^2^ > 2σ(*F* ^2^)] = 0.033
*wR*(*F* ^2^) = 0.084
*S* = 1.10
2944 reflections
182 parameters
H-atom parameters constrained
Δρ_max_ = 0.36 e Å^−3^
Δρ_min_ = −0.16 e Å^−3^
Absolute structure: Flack (1983 ▶), 1407 Friedel pairs
Flack parameter: 0.09 (9)

### 

Data collection: *SMART* (Bruker, 2007 ▶); cell refinement: *SAINT* (Bruker, 2007 ▶); data reduction: *SAINT*; program(s) used to solve structure: *SHELXS97* (Sheldrick, 2008 ▶); program(s) used to refine structure: *SHELXL97* (Sheldrick, 2008 ▶); molecular graphics: *SHELXTL* (Sheldrick, 2008 ▶); software used to prepare material for publication: *SHELXTL*.

## Supplementary Material

Crystal structure: contains datablock(s) global, I. DOI: 10.1107/S160053681201803X/tk5088sup1.cif

Structure factors: contains datablock(s) I. DOI: 10.1107/S160053681201803X/tk5088Isup2.hkl

Supplementary material file. DOI: 10.1107/S160053681201803X/tk5088Isup3.cml

Additional supplementary materials:  crystallographic information; 3D view; checkCIF report

## Figures and Tables

**Table 1:** Hydrogen-bond geometry (Å, °) *Cg*1 and *Cg*2 are the centroids of the C1–C6 and C9–C14 rings, respectively.

*D*—H⋯*A*	*D*—H	H⋯*A*	*D*⋯*A*	*D*—H⋯*A*
N2—H2*A*⋯O1^i^	0.86	2.12	2.921 (2)	156
N1—H1*A*⋯Cl1^ii^	0.86	2.94	3.680 (2)	145
N1—H1*B*⋯O1^iii^	0.86	2.19	2.978 (3)	151
O2—H2⋯N3	0.82	1.88	2.588 (3)	145
C5—H5⋯*Cg*2^iv^	0.93	2.92	3.473 (3)	120
C14—H14⋯*Cg*1^v^	0.93	2.83	3.641 (3)	147

Symmetry codes: (i) 
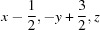
; (ii) 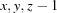; (iii) 
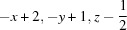
; (iv) 
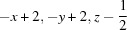
; (v) 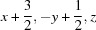.

**Fig. 1.**
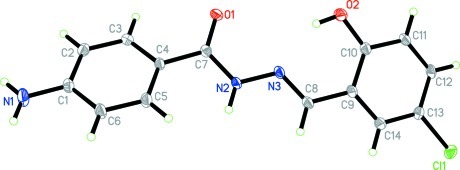
The molecular structure of the title compound, with displacement ellipsoids at the 30% probability level.

**Fig. 2.**
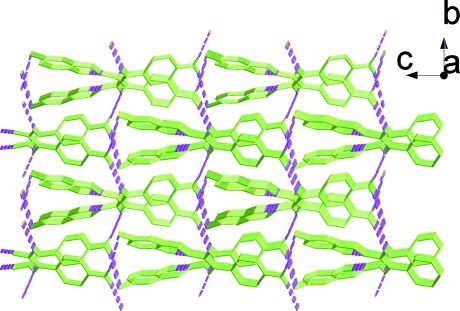
Crystal packing in the title compound where molecules are linked via N—H···O and N—H···Cl hydrogen bonds (dashed lines). Except for those involved in hydrogen-bonding interactions, H atoms have been omitted for clarity.

## supplementary crystallographic information

### Comment

Schiff bases are an important class of compounds in the medicinal and pharmaceutical fields and have been found to play a role in the development of coordination chemistry as they readily form stable complexes with most transition metals. These complexes show interesting properties, *e.g*. their ability to reversibly bind oxygen, catalytic activity in hydrogenation of olefins and transfer of an amino group, photochromic properties, and complexing ability towards toxic metals (Karthikeyan *et al.*, 2006; Khattab, 2005; Kucukguzel *et al.*, 2006). Recently, hydrazone Schiff base compounds (Cao, 2009; Yang, 2009; Zhou & Yang, 2010; Zhang *et al.*, 2009) derived from the reaction of aldehydes with hydrazines have been demonstrated to possess excellent biological activities, such as anti-bacterial, anti-convulsant, and anti-tubercular (Bernhardt & Caldwell *et al.*, 2003; Bernhardt & Chin *et al.*, 2005; Armstrong *et al.*, 2003). In order to explore new anti-bacterial materials, a new hydrazone derivatives was prepared and characterized. It can be seen that in addition to the presence of the typical functional group —CO—NH—N═ CH—, the compound has chloro, hydroxy and amino substituents, which may enhance its biological properties.

As shown in Fig. 1, the molecule displays an *E* configuration with respect to the C═N double bond. The dihedral angle between the two benzene rings is 41.3 (5)°. The bond lengths and angles are as expected for a compound of this type and agree with the other ligands belonging to the hydrazone series, mentioned above. The C8═N3 and C7═O1 bond lengths of 1.287 (3) and 1.238 (3) Å, respectively, conform to the values for double bonds. Whereas the C1—N1, C7—N2, C10—O2 and N2—N3 [1.367 (3), 1.370 (3), 1.355 (3) and 1.368 (3) Å, respectively] bond lengths correspond to a single bond. In the crystal packing, it is noted that amino H (H1A, H1B) and amide H2A are involved in forming intermolecular N—H···O and N—H···Cl hydrogen bonds (Fig. 2 and Table 1), linking the molecules into a three-dimensional supramolecular structure. In addition, neighbouring molecules are also interact through weak C—H···π stacking interactions, Table 1.

### Experimental

To a methanol solution (20 ml) of 5-chloro-2-hydroxybenzaldehyde (1 mmol, 0.157 g) and 4-aminobenzohydrazide (1 mmol, 0.151 g), a few drops of acetic acid were added. The mixture was refluxed for 2 h and then cooled to room temperature to give a yellow solution. Crystals of the title compound were formed by gradual evaporation of the solvent over a period of six days at room temperature.

### Refinement

H-atoms were placed in calculated positions (C—H = 0.93, O—H = 0.82 and N—H = 0.86-0.89 Å) and were included in the refinement in the riding model approximation, with *U*_iso_(H) set to 1.2*U*_eq_(C or N) and 1.5 *U*_eq_(O).

### Crystal data

**Table:**

C_14_H_12_ClN_3_O_2_	*F*(000) = 600
*M**_r_* = 289.72	*D*_x_ = 1.487 Mg m^−^^3^
Orthorhombic, *P**n**a*2_1_	Mo *K*α radiation, λ = 0.71073 Å
Hall symbol: P 2c -2n	Cell parameters from 6341 reflections
*a* = 9.3375 (16) Å	θ = 1.4–27.5°
*b* = 9.7194 (16) Å	µ = 0.30 mm^−^^1^
*c* = 14.214 (3) Å	*T* = 296 K
*V* = 1290.0 (4) Å^3^	Block, yellow
*Z* = 4	0.20 × 0.15 × 0.10 mm

### Data collection

**Table:**

Bruker SMART CCD area-detector diffractometer	2944 independent reflections
Radiation source: fine-focus sealed tube	2866 reflections with *I* > 2σ(*I*)
Graphite monochromator	*R*_int_ = 0.038
φ and ω scans	θ_max_ = 27.5°, θ_min_ = 2.5°
Absorption correction: multi-scan (*SADABS*; Sheldrick, 1996)	*h* = −11→12
*T*_min_ = 0.947, *T*_max_ = 0.970	*k* = −12→9
8516 measured reflections	*l* = −18→18

### Refinement

**Table:**

Refinement on *F*^2^	Secondary atom site location: difference Fourier map
Least-squares matrix: full	Hydrogen site location: inferred from neighbouring sites
*R*[*F*^2^ > 2σ(*F*^2^)] = 0.033	H-atom parameters constrained
*wR*(*F*^2^) = 0.084	*w* = 1/[σ^2^(*F*_o_^2^) + (0.0553*P*)^2^ + 0.2086*P*] where *P* = (*F*_o_^2^ + 2*F*_c_^2^)/3
*S* = 1.10	(Δ/σ)_max_ < 0.001
2944 reflections	Δρ_max_ = 0.36 e Å^−^^3^
182 parameters	Δρ_min_ = −0.16 e Å^−^^3^
0 restraints	Absolute structure: Flack (1983), 1407 Friedel pairs
Primary atom site location: structure-invariant direct methods	Flack parameter: 0.09 (9)

### Special details

**Table:**

Geometry. All esds (except the esd in the dihedral angle between two l.s. planes) are estimated using the full covariance matrix. The cell esds are taken into account individually in the estimation of esds in distances, angles and torsion angles; correlations between esds in cell parameters are only used when they are defined by crystal symmetry. An approximate (isotropic) treatment of cell esds is used for estimating esds involving l.s. planes.
Refinement. Refinement of F^2^ against ALL reflections. The weighted R-factor wR and goodness of fit S are based on F^2^, conventional R-factors R are based on F, with F set to zero for negative F^2^. The threshold expression of F^2^ > 2sigma(F^2^) is used only for calculating R-factors(gt) etc. and is not relevant to the choice of reflections for refinement. R-factors based on F^2^ are statistically about twice as large as those based on F, and R- factors based on ALL data will be even larger.

### Fractional atomic coordinates and isotropic or equivalent isotropic displacement parameters (Å^2^)

**Table:**

	*x*	*y*	*z*	*U*_iso_*/*U*_eq_	
Cl1	0.75084 (6)	0.97018 (6)	0.79216 (7)	0.03468 (17)	
O1	1.14409 (16)	0.65129 (17)	0.27805 (12)	0.0267 (4)	
O2	1.19222 (18)	0.8254 (2)	0.51735 (12)	0.0343 (4)	
H2	1.1593	0.8184	0.4641	0.051*	
N1	0.8561 (3)	0.6238 (2)	−0.12949 (16)	0.0377 (5)	
H1A	0.7980	0.6813	−0.1551	0.045*	
H1B	0.8867	0.5542	−0.1610	0.045*	
N2	0.94718 (19)	0.78349 (19)	0.30263 (14)	0.0224 (4)	
H2A	0.8682	0.8164	0.2816	0.027*	
N3	0.9907 (2)	0.8088 (2)	0.39290 (13)	0.0224 (4)	
C1	0.8999 (2)	0.6432 (2)	−0.03874 (17)	0.0262 (5)	
C2	0.9933 (3)	0.5508 (2)	0.00555 (18)	0.0270 (5)	
H2B	1.0272	0.4748	−0.0273	0.032*	
C3	1.0352 (2)	0.5718 (2)	0.09760 (17)	0.0255 (5)	
H3	1.0982	0.5103	0.1257	0.031*	
C4	0.9840 (2)	0.6846 (2)	0.14908 (16)	0.0220 (4)	
C5	0.8906 (3)	0.7754 (3)	0.10492 (17)	0.0286 (5)	
H5	0.8548	0.8503	0.1382	0.034*	
C6	0.8504 (3)	0.7562 (3)	0.01244 (17)	0.0308 (5)	
H6	0.7895	0.8193	−0.0161	0.037*	
C7	1.0335 (2)	0.7040 (2)	0.24690 (16)	0.0220 (4)	
C8	0.9037 (2)	0.8682 (2)	0.45000 (17)	0.0239 (5)	
H8	0.8149	0.8985	0.4294	0.029*	
C9	0.9471 (2)	0.8871 (2)	0.54757 (16)	0.0230 (4)	
C10	1.0863 (2)	0.8585 (2)	0.57817 (17)	0.0247 (5)	
C11	1.1199 (2)	0.8632 (2)	0.67355 (18)	0.0274 (5)	
H11	1.2124	0.8434	0.6930	0.033*	
C12	1.0177 (3)	0.8968 (2)	0.73943 (16)	0.0268 (5)	
H12	1.0402	0.8979	0.8031	0.032*	
C13	0.8807 (3)	0.9291 (2)	0.70915 (17)	0.0256 (5)	
C14	0.8452 (3)	0.9260 (2)	0.61485 (16)	0.0253 (5)	
H14	0.7534	0.9497	0.5959	0.030*	

### Atomic displacement parameters (Å^2^)

**Table:**

	*U*^11^	*U*^22^	*U*^33^	*U*^12^	*U*^13^	*U*^23^
Cl1	0.0388 (3)	0.0439 (3)	0.0213 (3)	0.0096 (2)	0.0028 (2)	−0.0028 (3)
O1	0.0207 (7)	0.0360 (8)	0.0235 (9)	0.0020 (6)	−0.0044 (6)	0.0024 (7)
O2	0.0268 (9)	0.0562 (11)	0.0198 (8)	0.0067 (8)	−0.0026 (7)	0.0004 (8)
N1	0.0550 (14)	0.0359 (11)	0.0221 (11)	0.0034 (10)	−0.0080 (10)	−0.0054 (9)
N2	0.0218 (8)	0.0301 (9)	0.0155 (8)	0.0004 (7)	−0.0036 (7)	0.0002 (7)
N3	0.0232 (9)	0.0287 (9)	0.0152 (8)	−0.0022 (7)	−0.0037 (7)	0.0020 (7)
C1	0.0289 (11)	0.0308 (12)	0.0188 (10)	−0.0055 (9)	−0.0003 (8)	0.0001 (9)
C2	0.0284 (11)	0.0270 (10)	0.0254 (11)	0.0000 (9)	0.0027 (9)	−0.0042 (10)
C3	0.0228 (10)	0.0289 (11)	0.0247 (12)	0.0021 (9)	−0.0020 (9)	−0.0010 (9)
C4	0.0227 (10)	0.0267 (10)	0.0166 (10)	−0.0023 (8)	−0.0013 (8)	−0.0002 (8)
C5	0.0374 (12)	0.0291 (11)	0.0195 (11)	0.0054 (9)	−0.0026 (9)	−0.0010 (9)
C6	0.0403 (14)	0.0317 (11)	0.0206 (11)	0.0065 (10)	−0.0077 (10)	0.0004 (9)
C7	0.0236 (10)	0.0239 (10)	0.0185 (11)	−0.0033 (8)	−0.0003 (9)	0.0029 (8)
C8	0.0235 (10)	0.0288 (11)	0.0195 (11)	−0.0007 (8)	−0.0047 (8)	0.0018 (9)
C9	0.0271 (11)	0.0239 (10)	0.0180 (10)	−0.0009 (8)	−0.0033 (8)	0.0010 (9)
C10	0.0263 (11)	0.0274 (10)	0.0205 (11)	0.0000 (8)	−0.0032 (9)	−0.0010 (9)
C11	0.0258 (11)	0.0325 (11)	0.0239 (12)	0.0019 (9)	−0.0093 (9)	−0.0012 (9)
C12	0.0360 (12)	0.0299 (11)	0.0146 (10)	0.0002 (9)	−0.0062 (9)	−0.0001 (9)
C13	0.0322 (12)	0.0248 (10)	0.0197 (11)	0.0011 (8)	0.0015 (9)	−0.0022 (9)
C14	0.0252 (11)	0.0293 (10)	0.0215 (11)	0.0019 (9)	−0.0033 (8)	0.0009 (9)

### Geometric parameters (Å, º)

**Table:**

Cl1—C13	1.741 (2)	C4—C5	1.392 (3)
O1—C7	1.235 (2)	C4—C7	1.479 (3)
O2—C10	1.355 (3)	C5—C6	1.381 (3)
O2—H2	0.8200	C5—H5	0.9300
N1—C1	1.367 (3)	C6—H6	0.9300
N1—H1A	0.8600	C8—C9	1.458 (3)
N1—H1B	0.8600	C8—H8	0.9300
N2—N3	1.369 (3)	C9—C10	1.399 (3)
N2—C7	1.370 (3)	C9—C14	1.403 (3)
N2—H2A	0.8600	C10—C11	1.393 (3)
N3—C8	1.287 (3)	C11—C12	1.378 (3)
C1—C6	1.396 (3)	C11—H11	0.9300
C1—C2	1.402 (3)	C12—C13	1.390 (3)
C2—C3	1.382 (3)	C12—H12	0.9300
C2—H2B	0.9300	C13—C14	1.382 (3)
C3—C4	1.402 (3)	C14—H14	0.9300
C3—H3	0.9300		
			
C10—O2—H2	109.5	C1—C6—H6	119.6
C1—N1—H1A	120.0	O1—C7—N2	121.3 (2)
C1—N1—H1B	120.0	O1—C7—C4	123.1 (2)
H1A—N1—H1B	120.0	N2—C7—C4	115.54 (19)
N3—N2—C7	117.90 (19)	N3—C8—C9	118.7 (2)
N3—N2—H2A	121.1	N3—C8—H8	120.6
C7—N2—H2A	121.1	C9—C8—H8	120.6
C8—N3—N2	118.93 (19)	C10—C9—C14	118.3 (2)
N1—C1—C6	120.1 (2)	C10—C9—C8	122.0 (2)
N1—C1—C2	121.5 (2)	C14—C9—C8	119.6 (2)
C6—C1—C2	118.4 (2)	O2—C10—C11	117.7 (2)
C3—C2—C1	120.6 (2)	O2—C10—C9	122.0 (2)
C3—C2—H2B	119.7	C11—C10—C9	120.4 (2)
C1—C2—H2B	119.7	C12—C11—C10	120.9 (2)
C2—C3—C4	120.8 (2)	C12—C11—H11	119.6
C2—C3—H3	119.6	C10—C11—H11	119.6
C4—C3—H3	119.6	C11—C12—C13	119.0 (2)
C5—C4—C3	118.3 (2)	C11—C12—H12	120.5
C5—C4—C7	122.7 (2)	C13—C12—H12	120.5
C3—C4—C7	118.9 (2)	C14—C13—C12	121.2 (2)
C6—C5—C4	121.0 (2)	C14—C13—Cl1	119.69 (19)
C6—C5—H5	119.5	C12—C13—Cl1	119.13 (18)
C4—C5—H5	119.5	C13—C14—C9	120.3 (2)
C5—C6—C1	120.8 (2)	C13—C14—H14	119.9
C5—C6—H6	119.6	C9—C14—H14	119.9
			
C7—N2—N3—C8	−171.4 (2)	N2—N3—C8—C9	176.14 (19)
N1—C1—C2—C3	−179.5 (2)	N3—C8—C9—C10	8.1 (3)
C6—C1—C2—C3	−0.2 (4)	N3—C8—C9—C14	−167.4 (2)
C1—C2—C3—C4	0.9 (4)	C14—C9—C10—O2	−177.5 (2)
C2—C3—C4—C5	−0.5 (3)	C8—C9—C10—O2	6.9 (3)
C2—C3—C4—C7	−179.1 (2)	C14—C9—C10—C11	2.7 (3)
C3—C4—C5—C6	−0.7 (4)	C8—C9—C10—C11	−172.9 (2)
C7—C4—C5—C6	177.9 (2)	O2—C10—C11—C12	179.7 (2)
C4—C5—C6—C1	1.4 (4)	C9—C10—C11—C12	−0.4 (4)
N1—C1—C6—C5	178.3 (2)	C10—C11—C12—C13	−1.4 (4)
C2—C1—C6—C5	−1.0 (4)	C11—C12—C13—C14	1.0 (4)
N3—N2—C7—O1	3.3 (3)	C11—C12—C13—Cl1	179.46 (18)
N3—N2—C7—C4	−177.71 (18)	C12—C13—C14—C9	1.3 (4)
C5—C4—C7—O1	−158.2 (2)	Cl1—C13—C14—C9	−177.17 (17)
C3—C4—C7—O1	20.4 (3)	C10—C9—C14—C13	−3.1 (3)
C5—C4—C7—N2	22.8 (3)	C8—C9—C14—C13	172.6 (2)
C3—C4—C7—N2	−158.6 (2)		

### Hydrogen-bond geometry (Å, º)

Cg1 and Cg2 are the centroids of the C1–C6 and C9–C14 rings, respectively.

**Table:**

*D*—H···*A*	*D*—H	H···*A*	*D*···*A*	*D*—H···*A*
N2—H2*A*···O1^i^	0.86	2.12	2.921 (2)	156
N1—H1*A*···Cl1^ii^	0.86	2.94	3.680 (2)	145
N1—H1*B*···O1^iii^	0.86	2.19	2.978 (3)	151
O2—H2···N3	0.82	1.88	2.588 (3)	145
C5—H5···*Cg*2^iv^	0.93	2.92	3.473 (3)	120
C14—H14···*Cg*1^v^	0.93	2.83	3.641 (3)	147

Symmetry codes: (i) *x*−1/2, −*y*+3/2, *z*; (ii) *x*, *y*, *z*−1; (iii) −*x*+2, −*y*+1, *z*−1/2; (iv) −*x*+2, −*y*+2, *z*−1/2; (v) *x*+3/2, −*y*+1/2, *z*.

## References

[bb1] Armstrong, C. M., Bernhardt, P. V., Chin, P. & Richardson, D. R. (2003). *Eur. J. Inorg. Chem.* pp. 1145–1156.

[bb2] Bernhardt, P. V., Caldwell, L. M., Chaston, T. B., Chin, P. & Richardson, D. R. (2003). *J. Biol. Inorg. Chem.* **8**, 866–880. 10.1007/s00775-003-0486-z 14564555

[bb3] Bernhardt, P. V., Chin, P., Sharpe, P. C., Wang, J. Y. C. & Richardson, D. R. (2005). *J. Biol. Inorg. Chem.* **10**, 761–777. 10.1007/s00775-005-0018-0 16193304

[bb4] Bruker (2007). *SMART* and *SAINT* Bruker AXS Inc., Madison, Wisconsin, USA.

[bb5] Cao, G.-B. (2009). *Acta Cryst.* E**65**, o2415. 10.1107/S1600536809035740 PMC2970190 21577874

[bb6] Flack, H. D. (1983). *Acta Cryst.* A**39**, 876–881.

[bb7] Karthikeyan, M. S., Prasad, D. J., Poojary, B., Bhat, K. S., Holla, B. S. & Kumari, N. S. (2006). *Bioorg. Med. Chem.* **14**, 7482–7489. 10.1016/j.bmc.2006.07.015 16879972

[bb8] Khattab, S. N. (2005). *Molecules*, **10**, 1218–1228. 10.3390/10091218 PMC6147684 18007388

[bb9] Kucukguzel, G., Kocatepe, A., De Clercq, E., Sahi, F. & Gulluce, M. (2006). *Eur. J. Med. Chem.* **41**, 353–359. 10.1016/j.ejmech.2005.11.005 16414150

[bb10] Sheldrick, G. M. (1996). *SADABS* University of Göttingen, Germany.

[bb11] Sheldrick, G. M. (2008). *Acta Cryst.* A**64**, 112–122. 10.1107/S0108767307043930 18156677

[bb12] Yang, D.-S. (2009). *Acta Cryst.* E**65**, o2864. 10.1107/S1600536809043840 PMC2971021 21578451

[bb13] Zhang, M.-J., Yin, L.-Z., Wang, D.-C., Deng, X.-M. & Liu, J.-B. (2009). *Acta Cryst.* E**65**, o508. 10.1107/S1600536809002165 PMC2968684 21582171

[bb14] Zhou, C.-S. & Yang, T. (2010). *Acta Cryst.* E**66**, o365. 10.1107/S1600536810001303 PMC2979760 21579789

